# The buzz about honey-based biosurveys

**DOI:** 10.1038/s44185-024-00040-y

**Published:** 2024-04-17

**Authors:** Paton Vuong, Anna Poppy Griffiths, Elizabeth Barbour, Parwinder Kaur

**Affiliations:** UWA School of Agriculture & Environment, University of Western Australia, Perth, Australia

**Keywords:** Biotechnology, Microbiology

## Abstract

Approximately 1.8 million metric tonnes of honey are produced globally every year. The key source behind this output, the honey bee (*Apis mellifera)*, works tirelessly to create the delicious condiment that is consumed worldwide. The honey that finds its way into jars on store shelves contains a myriad of information about its biogeographical origins, such as the bees that produced it, the botanical constituents, and traces of other organisms or pathogens that have come in contact with the product or its producer. With the ongoing threat of honey bee decline and overall global biodiversity loss, access to ecological information has become an key factor in preventing the loss of species. This review delves into the various molecular techniques developed to characterize the collective DNA harnessed within honey samples, and how it can be used to elucidate the ecological interactions between honey bees and the environment. We also explore how these DNA-based methods can be used for large-scale biogeographical studies through the environmental DNA collected by foraging honey bees. Further development of these techniques can assist in the conservation of biodiversity by detecting ecosystem perturbations, with the potential to be expanded towards other critical flying pollinators.

## Introduction

Global declines in honey bee populations have become a growing concern within the apiary industry and beyond^1,2^. COLOSS (https://coloss.org/) is an association that was formed in response to honey bee colony losses, with its core projects aiming to provide monitoring and reporting standards for understanding colony losses. The latest global collation of COLOSS-based surveys across 37 countries reported that the winter loss rates for 2019–2020 varied between 7.4% and 36.5%, with the overall average loss higher than those reported in the previous year^3^. Honey bees provide vital pollination services to a range of commercial crops, such as almonds, pome fruit and rape seed^4^, with roughly 90% of commercial pollination carried out by managed *Apis mellifera* populations^5^. Their contribution of honey products is also significant to agricultural output, with the honey market expected to reach a predicted value of 11.88 billion USD by the year 2028^6^. Honey bees in both managed and wild populations also act to maintain plant biodiversity in their foraging regions by facilitating fertilisation, a critical process in many ecosystems^7^. Honey bees are referred to as superorganisms, whereby an entire colony is regarded as a collective organism, meaning that any ecological shifts may cascade across the entire hive^8^. Given their interconnected nature with the surrounding biodiversity, honey bees and their products could provide a focal point for uncovering ecological perturbations that may threaten the health of colonies and their ecosystems^9^. Consequently, the development of an approach that can provide large-scale biosurveys for conservation purposes is critical for the safeguarding of bees and their associated biodiversity.

Honey acts like a living library, akin to a store of information from the surrounding environment^10^ and the books deposited are the genetic materials collected from the journeys of thousands of honey bees^11,12^. DNA-based analyses can delve into this “honey-pot” of collected information, bringing forth insights into ecological interactions between honey bees and their environment^13^. The DNA detection methods for detecting biodiversity in environmental samples may be key to addressing the issues faced by the apiary industry by providing ecological assessments through the identification of invasive and endemic species^14,15^. This review discusses existing DNA analysis techniques and how they can be optimised to extract genetic information from honey samples. We explore DNA-based approaches initially developed for the apiary industry and discuss their potential for further applications, such as providing a wide-area biosurvey tool, understanding biodiversity networks, detecting ecological perturbations, and elucidating the co-evolution of symbiotic taxa through the analyses of honey.

## Unearthing biodiversity: application of DNA-based techniques in honey

The classification of taxa is vital to understanding biodiversity. Traditional identification methods, especially in plants, have relied largely on the comparison of morphological and anatomical attributes^16^. In honey samples, the morphological classification of pollen—termed melissopalynology—is an established technique used to identify the floral plants from which it was derived^17^. However, morphological-based identification of taxonomy is problematic, confounded by individual opinion and lack of general consensus, and can have difficulty distinguishing species with similar attributes^18,19^. Melissopalynology in honey samples also has questionable reliability, especially when classifying pollen from highly diverse ecosystems or those with an overlap in flowering seasons of floral species^20^. The development of DNA-based techniques has provided an increasingly precise, scalable and universally applicable classification method^21^. DNA analysis has led to advances in taxonomy through the redefinition of species delimitations, being unaffected by factors such as convergent evolution, which confined morphological-based classification in the past^22,23^.

Environmental DNA is the genetic material obtained directly from environmental samples and contains broad information from organisms within the sampling location^24^. A variety of DNA-based techniques have elucidated a spectrum of species from various environments, such as habitat samples (e.g. soil and water), or material that would accumulate traces of DNA from the (micro)biota of the surrounding environment (e.g. tissue samples, faecal matter, mucus, and digestive contents)^15^. Traditional methods of DNA-based analyses involved the PCR amplification of known target DNA, which served as a simple method for detecting the presence of the DNA target(s) of interest (qualitative PCR), or both the detection and quantification of the DNA target(s) within a given sample (quantitative or real-time PCR)^25^. In honey, these traditional DNA-based methods were used to detect or quantify the presence of species of interest, such as to confirm honey bee species, screen for pathogens, or ascertain claimed floral origins^13^. However, due to the focused and relatively small-scale capacity of qualitative and quantitative PCR, they are not suitable for the wide and all-encompassing approach needed to elucidate biodiversity on an extensive scale.

**Fig. 1:**
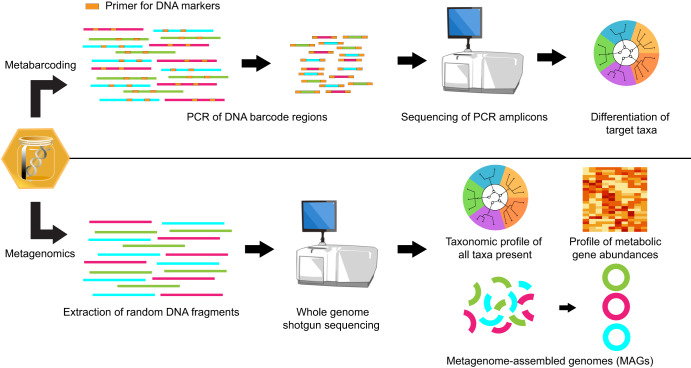
Simplified workflow of current DNA-based methods for analysis of (micro)organisms within environmental samples, using honey as an example. Metabarcoding compares target gene regions to differentiate different taxa. Metagenomics looks at all genetic information as a collective, allowing for additional information to be gleaned from the sequence data.

High-throughput sequencing has enabled the ability to generate large-scale datasets, providing deeper insights into the (micro)organisms from environmental samples^26,27^ (Fig. 1). Although advances have been made for DNA-based analysis applications in honey samples^13^, there has yet to be an industry standard for this testing methodology. The following section explores the current DNA-based approaches (Table 1) and their applicability and limitations in honey-based analysis.

**Table 1:** A comparison of DNA-based techniques

Method	Advantages	Limitations
Metabarcoding^13,29,32,33^	Widely trialled for biodiversity and ecological assessment. Provides cost and time-effective analysis. Successful results with low-biomass input	Extensive reference databases are required to find suitable marker regions within candidate genomes. Species identification is reliant on curated marker catalogues. Output is limited to barcode regions. PCR introduces amplification bias, resulting in skewed abundance data. PCR hindered by contaminants, additives, and degraded DNA
Metagenomics^36,38–40^	Sequence data is all-encompassing. No reliance on PCR. Taxa recovery is not limited by primers. Novel species identification.	Reference genomes are needed for classification. Existing reference libraries are limited and often incomplete, especially for eukaryotes. Analysis may be expensive and time-consuming.

### Metabarcoding/amplicon sequencing

A popular approach in honey-based biodiversity studies is the use of metabarcoding, a DNA-based method that targets specific DNA markers (i.e., gene regions present in multiple species) with universal primers^11,13,28^. While species are highly conserved in the flanking regions targeted by the primers, the sequences between are highly variable, allowing distinction and classification of organisms^29^. PCR amplification of DNA markers and subsequent sequencing produces classifiable data that is used for taxonomic analysis^26,29^. Table 2 is a non-exhaustive list of common universal DNA markers used to distinguish genetic material of entomological, botanical, and microbial origins. Metabarcoding has been trialled across various ecosystems and environmental samples for use in biodiversity surveys, and has shown to be a cost and time-effective ecological assessment tool^29^.

**Table 2:** Universal genetic markers used for DNA-based analysis in apiary studies

DNA marker categories	Target gene or genetic region	Applications in apiary biomonitoring and biodiversity tracing
Cytochrome c oxidase complex	Cytochrome oxidase I (COI)	COI has been the predominant metabarcoding marker for distinguishing between metazoans species^124^. As such, it is mainly utilised for determining entomological sources, such as differentiation of honey bees from different geographical regions^125^, as well as traces of Hemiptera species as honeydew sources^72^.
Ribosomal subunits	18 S ribosomal RNA (18 S) 16 S ribosomal RNA (16 S)	18 S has been widely used as a universal primer for all eukaryotes in environmental samples^126^, but honey-based studies appear to prefer more biologically relevant targets such as chloroplast genes for plants and COI for metazoans. In comparison, 16 S rRNA remains the most widely used universal primer for prokaryotes across different environmental samples^127^ and has been used to identify bacterial species in honey to identify bee gut microbiota, microbes via interactions with the surrounding environment, as well as potential pathogens^11^.
Internal transcribed spacer	ITS1 ITS2	Markers for both ITS1 and ITS2 regions have been used extensively to classify both fungal^128^ and plant^129^ species in DNA-based studies. ITS2 alongside *rbcL* was able to successfully identify over 900 plant species from honey samples^130^, and when compared to a melissopalynological approach was able to conclude the same botanical species in an easier fashion^97^. Fungal communities in honey were tested using ITS2 to ascertain microbial quality control^131^, and via the complete ITS region to delineate honey samples from different geographical regions^11^.
Plant chloroplast	Ribulose bisphosphate carboxylase (*rbcL)* Chloroplast tRNA gene intron (*trnL*) Maturase K (*matK*) trnH-psbA intergenic spacer region (*trnH-psbA*)	Plastid-based markers from the plant chloroplast are used to differentiate species, although there are still debates on which provides optimal plant identification, especially for use in honey samples^132^. Many honey sample studies use more than one set of markers, with plastid-based markers such as trnH-psbA^133^, *matK*^134^, *rbcL*^130^ *or trnL*^135^ used alongside the nuclear ribosomal ITS2 for increased resolution in delineating species.

Finding suitable DNA markers requires extensive reference databases, with the chief repositories of DNA marker sequences being Barcode of Life Data System (BOLD)^30^ and Genbank^31^. The sequences provided by these databases are critical for collating DNA barcodes from loci of interest, allowing the subsequent creation of universal primers that span across multiple taxa for broad range identification^32^. The collective and continuous submission of sequence data is essential for the expansion of these databases, as the resolution of taxonomic information from honey samples is reliant on having close matches to the reference sequences for relevant species^33^. However, the need for proper curation and inclusion of relevant species is vital for the provision of an effective reference database for use in metabarcoding^34^. As such, the creation of custom databases for honey bees and their associated biodiversity would greatly improve and expedite the analytical process. The HoloBee Database (https://data.nal.usda.gov/dataset/holobee-database-v20161) is a prime example of a collection of barcoding loci and genome assemblies of (micro)organisms associated with honey bees. The HoloBee database was released in 2016, with annual updates projected, but the project appears to have stopped receiving updates in 2019. BEExact^35^ is another honey-bee-related database that is currently updated but is limited to prokaryotes as it only contains 16 S rRNA gene sequences. There remains a need for curated and up-to-date multi-kingdom reference information for honey bee-associated biodiversity, which is key for unlocking the collective genetic information present within the honey metagenome.

### Metagenomics/whole genome shotgun sequencing

Another approach for the study of DNA from environmental samples is metagenomics. In contrast to the marker-centric approach in metabarcoding, metagenomics uses shotgun sequencing, which sequences all DNA within a sample in a random but all-encompassing manner^36^. The reads generated from sequencing data can then be aligned to gene, protein, and genome reference sequences, enabling both functional and taxonomic profiling^26^. As shotgun sequences can be assembled into longer contigs, they can provide higher resolution during classification and annotation through better coverage when aligned against genomes of reference species^26,36^. Additionally, assembled metagenomic data can be used to recover metagenome-assembled genomes (MAGs), which are genomes formed from collections of similar sequences from environmental samples^37^. The study of MAGs allows culture-independent discovery of novel species and has been key in expanding the knowledge of the tree of life^38^. The use of sequence assembly and MAG recovery from honey samples would assist in building much-needed reference data for the classification of taxa from the honey-based metagenome.

In comparison to the extensive use of metabarcoding, the metagenomic approach has seen relatively limited use in studies on honey samples^13^. However, the few studies utilising shotgun sequencing in honey samples were able to identify taxa across different kingdoms of life from the metagenomic reads generated^9,39,40^. Whilst the primers metabarcoding uses are termed ‘universal’, they are limited to the group of (micro)organisms they were designed for, resulting in many taxa being excluded^40^. The metagenomic approach is not restricted in this fashion and can include data from all organisms that interact with bee colonies, including often overlooked creatures like rodents and other mammals^39^. Although metagenomics provides a promising alternative to the metabarcoding approach, there are many challenges within DNA-based methods that need to be overcome before either can undergo widespread utilisation as standard assays for honey sampling.

### Challenges and progress in DNA-based analysis methods

Honey presents a challenging substance for DNA extraction. Commercial honey may undergo processing that degrades and damages DNA^13^, and plant material embedded within the honey contains pectin, polyphenols, polysaccharides, and xylan, which have inhibitory effects on DNA polymerase leading to issues during PCR^41^. Adequate disruption methods such as bead beating is crucial to properly process the pollen found in honey as the pollen wall contains sporopollenin, a highly robust and recalcitrant polymer^42^. A paper by Soares et al. 2023 presented a comprehensive list of commercially available and alternative extraction protocols for DNA extraction targeting various biological sources from honey and included additional information like quality parameters such as yield and A260/A280 ratios, as well as the intended downstream DNA identification method^13^. Although commercial extraction kits provide ease of use, they may introduce bias in the extraction process as they are often optimised for specific tissue types (e.g. plant material). Contemporary extraction methods using cetyltrimethylammonium bromide and sodium dodecyl sulphate may reduce this form of bias, but they are often cumbersome and can require the use of hazardous chemicals^13^. Galanis et al. (2022) developed and evaluated a simple NaOH-based extraction method against a commercial plant-specific column-based kit for use in shotgun metagenomics of honey samples^9^. The NaOH-based extraction method was able to uncover a broader range of taxa from different kingdoms of life, expanding the capabilities of metagenomic analyses to cover both authentication and pathogen detection^9^. Further development and evaluation of nucleic acid extraction procedures are key to delving into the full spectrum of taxa present within the accumulated DNA in honey samples.

The PCR process required for metabarcoding is prone to amplification biases where primers may overamplify certain groups of taxa or non-target taxa^43^. Universal primers can also introduce bias to due to mismatches, particularly when distantly related species are included^43^. A technical review by Bohmann et al. 2021 delves further into the biases associated with DNA metabarcoding studies and offers strategies for designing experimental workflows (such as advice on sample-specific labelling and library preparation techniques) to produce robust data from environmental sampling^44^. Although shotgun sequencing is not dependent on PCR, the method relies on draft or complete reference genomes for annotation, with issues in classification rates occurring due to the lack of suitable reference sequences^9,39^. Similarly, metabarcoding approaches also have similar issues when attempting to identify species with poor levels of representation within reference databases^29^, indicating a need for further genetic surveys of both the natural and engineered environment. Naturally, the expansion of existing reference databases would improve the accuracy of DNA-based techniques for all environmental studies, not just honey-based sampling^38^. Due to the nature of its production, honey acts as a panoptic medium containing the ecological information of a multi-kingdom conglomerate^40^. Intrinsically, honey acts as a “DNA depot” and sequencing honey could provide a large range of genetic data from a relatively small and condensed sample, providing a less taxing alternative to traditional biodiversity survey methods^45^. As such, exploration into the honey metagenome provides a twofold benefit—the surveying of the collective taxa of the ecosystem, as well as the recovery of reference information through genome-resolved metagenomics.

The recovery of MAGs has been predominantly optimised and utilised for microorganisms (chiefly prokaryotes), but methods have been lacking for the retrieval of eukaryotic organisms^46^. There is an ongoing effort to expand the scope of available eukaryotic reference genomes due to their potential to aid studies in conservation and biodiversity^47^. The limited amount of eukaryotic reference genomes is due to a combination of expensive sequencing and assembly costs^48^, as well as a lack of extensive support in methodology and tools for eukaryotic MAG recovery from metagenomic data^49^. However, the availability of newer long-read sequencing platforms has improved the recovery of eukaryotic genomes and marker genes from metagenomic data through higher sequence resolution from the increased coverage of genomic sequences^50^. Long-read data from a mixed species pollen metagenome was able to be matched with shallow, low-coverage short-read reference sequences from known plants, showing a potential avenue of cost-effective botanical classification for honey samples^48^. In addition, the costs of high-throughput sequencing have constantly fallen, and long-read platforms will inevitably provide an avenue for non-microbial metagenomics in the near future^51^.

Further development of genome-resolved metagenomics provides an opportunity to expand our knowledge of the eukaryotic branches of the tree of life^46^. Obtaining eukaryotic MAGs from honey samples in this way would provide access to a vast amount of genomic information. Admittedly, the comparative volume of eukaryotic DNA would likely be limited in honey samples when compared to direct extraction from a tissue sample. However, access to a collated volume of draft or partial genomes would remain invaluable, providing a summary of the eukaryotic biodiversity present in the ecosystem of interest^52^. The foraging behaviour of honey bees enables widespread sampling, and the application of DNA-based analysis provides the tools needed to unravel the trove of ecological information stored within resulting samples of honey^9^.

The rapid growth of biological data has meant that there is a growing need to properly curate, facilitate access and update the troves of information^53^. The vast data that could be harvested from honey-based biosurveys would account for little if the data was unable to be properly utilised or disseminated. Responsible sharing, proper management of developing data and standardised reporting of metadata are required for the rapid access and efficient parsing of biological data^54^. One of the best guidelines for equitable data provision is the FAIR Principles (https://www.go-fair.org/fair-principles/) for Findability, Accessibility, Interoperability, and Reuse of digital information and assets. The FAIR Principles recommends that data should be made openly available, with actionable and properly referenced (meta)data that can be interacted with minimal human input, such as from AI or machine learning systems. The information from honey biosurveys should be easily available to all and any who need it and be provided in a future-accessible form that can be actioned by AI-based processing when data volumes inevitably grow beyond feasible analyses by human users.

## Beekeeping, biogeography and beyond

**Fig. 2:**
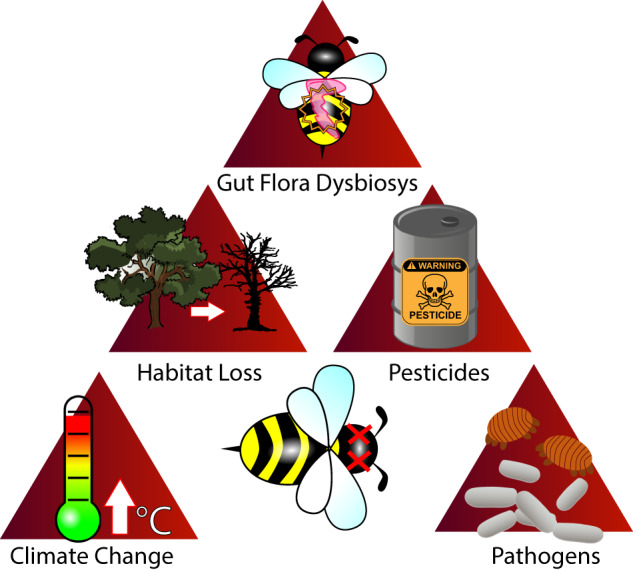
Visual summary of purported causes resulting in honey bee population declines. Honey bees experience numerous sources of ecological interaction and are highly sensitive to perturbations within the environment they reside in.

A chief issue faced by honey bees is population decline, and a comprehensive understanding of contributing causes is vital to combat this prevalent problem^55,56^ (Fig. 2). In addition to the economic value they provide, honey bees act as cornerstone species in many ecosystems, and global decline in populations are a threat to both pollinators and flowering plants alike^57,58^. DNA-based analysis of honey samples was introduced as a method of product authentication and biosecurity through the detection of botanical and entomological signatures^59,60^. However, the DNA-based approaches also provide a tool to monitor the potential factors of decline through revealing the multitude of ecological interactions experienced by honey bees. The collective DNA found in honey has the potential to be a platform to elucidate the wider biodiversity of the ecosystem it was derived from. As such, honey provides a potential platform to elucidate the wider array of lifeforms within an ecosystem, by the decoding of DNA accumulated by bees during its production.

### Product authentication drives access to biodiversity

The foundation of DNA-based methodologies in honey developed from a need to authenticate product origins and monitor the health of honey bee colonies^13^. Considered a lucrative and premium food product, honey is subject to high rates of adulteration, which includes the addition of cheap, commercially produced sugar syrups and mislabelling of geographic origins^61,62^. Historically, melissopalynology has been used to determine the geographical and botanical origin of honey by carrying out various honey pollen analyses^17^. However, due to the difficulty of melissopalynology techniques alongside their questionable effectiveness, spectrometry and chromatography methods have also been employed more recently to determine honey origin^63,64^. Numerous chemical markers for authentication have been identified, such as oligosaccharides to confirm honey purity and nectar sources^64^, as well as phytochemical composition (volatile compounds, phenolic compounds, carbohydrates and nitrogen-containing compounds) for confirming botanical and geographical origin^65^. Unfortunately, this approach also faces issues as the chemical makeup of honey is dependent on other external factors such as beekeeping husbandry, storage conditions, processing influences, etc., making the characterisation of chemical markers in honey unreliable or confounding in some cases^64,65^. To overcome the issues with current origin-related adulteration detection processes, researchers explored alternative approaches by utilising DNA-based techniques.

Honey contains a variety of genetic material from plants, fungi and bacteria that can be used to determine the region of origin^11^. DNA-based molecular techniques have a greater resolution in tracing pollen contained in honey samples, with DNA tracing able to classify the pollen to the genera level^11^, whereas morphological analyses struggle to identify past the family level^17,66^. DNA-based techniques can also circumvent the need for detectable levels of pollen^11^, which would allow plants with nectar, but low or absent pollen, to be detected^67,68^. Botanical tracing via DNA-based methods can even detect plant-derived compounds, with studies showing the ability to detect the presence of rice molasses^69^ and corn syrup^70^ from adulterated honey samples. Entomological data can also be recovered using DNA-based methods, which can distinguish the species of bee involved with the production of the honey sample^60,71^. This is crucial in authenticating honey that is advertised as “honey from native species” such as *Melipona beecheii*, instead of the larger colony-forming honey bee *A. mellifera*^68^. Genetic signatures from plant-sucking insects (order Hemiptera) were also detected in honey, both from honeydew and botanical honey, enabling potential insights into the inter-entomological foraging behaviour of honey bees^72^. The development of broad-range (micro)organism detection in product authentication has provided the potential for honey to act as a holistic sampling medium for large-scale multi-kingdom ecological studies.

### Colony health monitoring uncovers ecological interactions and relationships

Honey bees face a wide range of pathogens which vary in prevalence between honey bee species, as well as across geographical locations. Studies using molecular detection methods have made headway into the genotyping and genomic sequencing of honey bee pathogens, expanding the capacity of DNA-based detection and differentiation across target species^73^. Honey has been proposed as an environmental proxy to evaluate the health of bee colonies, with studies developing successful DNA-based methods for detecting various honey bee pathogens from honey samples^9,74–76^. Honey bee pests remain a persistent threat, and the comprehensive and non-invasive testing provided by DNA-based methods on honey samples could provide a vital tool for early detection and seasonal monitoring of pathogenic species^9^. Using honey as a testing medium allows traceability due to its wide-reaching distribution network as a commercial good, and DNA-based techniques may provide essential epidemiological and biogeographical information that could be utilised for biosurvey efforts^74^.

The ability to investigate the bee gut microbiota from honey samples may serve as an additional tool for monitoring the health of bee colonies^8,9,77^. The gut microbiota of adult honey bees are observed to be low complexity communities, with the composition of the microbial communities influenced by diet^78^ as well as through social interactions with other members of the hive^79,80^. The bee gut microbiota has been demonstrated to influence social interactions between bees^81^ as well as neurological and metabolic activity^82^. In addition, microbiota plays an essential role in maintaining the overall health of bees^78,83,84^, with disruption of the gut community resulting in increased mortality rates^85^ and vulnerability to viral infections^86^. The ability to monitor ecological drivers of bee health through DNA-based methods can provide insights for developing strategies to safeguard the well-being of honey bee populations^9^.

Aside from bee-related taxa, honey was also found to contain genetic information from animal- and plant-associated microorganisms containing both beneficial and pathogenic species^77^. This presents a critical avenue to explore and monitor the environmental microbiome for deleterious changes that could threaten biodiversity within an ecosystem of interest^87^. In addition, this confluence of (micro)organisms could unravel the dynamics of plant-^88^ and bee-microbe^81^ co-evolution and symbiosis and help determine whether there are common microbial drivers within the combined hologenome^39^. In the current era where global perturbations via anthropogenic activity are a constant source of ecological disruption, understanding the critical balance between host-microbial-environmental interactions is vital for preventing biodiversity loss^87,88^

### Honey bee foraging and biogeography

**Fig. 3:**
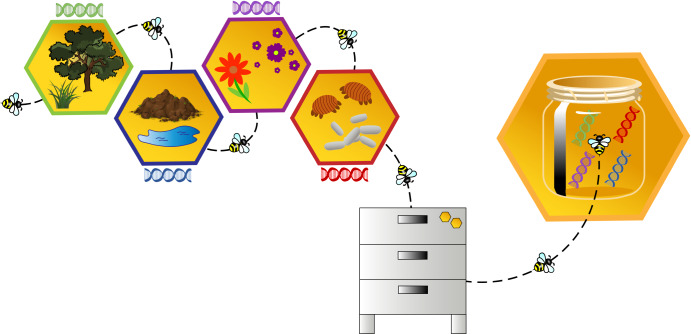
Depiction of bee foraging and the accumulation of genetic information from various sources. Honeybees collect a variety of biological material during foraging expeditions, which is subsequently captured within the honey produced by the hive.

Developing molecular techniques to utilise honey as a traceable medium provides a potential avenue to survey the ecological interactions between honey bees, their surrounding environment, and its associated biodiversity (Fig. 3). Honey bees are natural passive bioaccumulators, with the fine hairs on their bodies readily picking up material encountered during foraging trips in the greater environment^89^. These “records” of ecological interactions are subsequently deposited into the hive products—wax, propolis, and honey—providing a library of physicochemical and biological information about the surrounding environment^12,89^. The honey bee’s average flight distance is 2 km, servicing a ~12 km^2^ area around the colony^89^. A foraging bee can take upwards of ~13 trips per day and visits a wide range of ecological sites collecting pollen, water, nectar or propolis^90^. As colonies can contain an average of 3000 to 50,000 adult bees, with a quarter to a third of these members being foragers^91^, honey bees are able to provide a high volume and variety of sampling site coverage within their foraging area.

Biogeography explores the global distribution of biodiversity by studying how underlying ecological, spatial and temporal factors drive the dissemination and evolution of species^92^. Understanding the distribution dynamics of taxa is crucial, especially when biogeography is used in tandem with conservation projects, such as uncovering invasive species, monitoring biodiversity loss or directing efforts in ecological and/or species restoration^93^. Geogenomics is an emerging field that uses large-scale sequencing data to discern biogeographical patterns via genetic information^94^. The field of geogeonomics faces challenges in heterogeneous habitats, where high numbers of ecological interactions require interdisciplinary expertise to unravel the complex biogeography within^95^. The sampling power of honey bees via their extensive foraging behaviour can give crucial insights into seasonal or regional preferences of the bee diet and key pollination targets from visited flowering plant species^28,96,97^. As such, honey can serve as a focal medium for geogenomics, with the potential to elucidate biogeography through ecological interactions within and between honey bees, as well as their surrounding environment^39^. The honey-based biosurvey approach enables the surveillance of critical and cornerstone taxa—plants, pollinators, and their associated microbiota—within a geological region of interest.

### Advancing conservation and ecological understanding of biodiversity through honey sampling

Bees act as impromptu surveillance tools through the interaction of the surrounding flora, with the collected genetic material ultimately deposited into the honey reserves^11,39^. Climate change threatens plant biodiversity^98,99^, with remote sensing proposed as a method to monitor large-scale changes in diversity^100,101^. However, these broad vegetation surveying methods often see a reduction in species-based resolution in order to provide mass coverage^102^. DNA-based information from honey bee foraging may help supplement traditional surveys, with DNA from honey providing better detection of flowering species^33^.

Collective information on the environmental microbiota within honey may also present a solution to address the limited information on the status of global microbial biodiversity^103^. The phyllosphere consists of the aboveground portion of the plants, and the phyllospheric microbiome plays a vital role in plant health^104^. DNA-based methods were able to detect plant species, as well as phytopathogenic microbes from pollen collected from beehives, presenting a potential for honey to act as a botanical biosurveillance tool^105^. Furthermore, microbial sequence data has become a key factor in advancing biotechnology^106^, with the microbiome of the phyllosphere containing a cache of species producing bioproducts with plant growth, biological control, and pharmaceutical applications^104^. Large-scale sequencing of the honey metagenome has the capacity to add additional value on top of product authentication and biodiversity monitoring by providing resources for developing sustainable bioeconomic industries^106^.

Honey has also been suggested as a biomonitoring source for tracking the effects of environmental contaminants and anthropogenic activities^12^. Biological contaminants, including pathogenic bacteria and genetically modified organisms, are known to accumulate in honey. Thus the substrate could act as an early warning system for potential threats to both honey bee and human health^107^. In addition, the safety of pollinators within genetically modified agro-ecosystems is a potential concern, and the monitoring of genetically modified plant DNA in honey could provide insights into risk assessment studies of pollinator health^108^. Allergic reactions to artisanal or raw honey are due partly to the presence of pollen from plants, especially from certain families such as Asteraceae, and detecting botanical DNA from allergenic taxa of concern in honey can help provide warnings for both food safety and environmental allergen forecasts^109^. The use of honey biomonitoring for biological sources of concern has the potential to assist ecological assessments and management, as well as safeguard public health.

Due to the cheap, self-sustaining nature of beekeeping, there has been a recent movement proposing the utilisation of honey bee colonies as biosurveillance tools for environmental health^12,89,110^. As honey bees are one of the most commonly domesticated animals, there have also been initiatives to include backyard and other non-commercial beekeepers in biosurvey initiatives^111^. Although a non-traditional environment in ecological studies, the inclusion of backyard beehives can help explore the urban metagenome, with honey from urban hives yielding information on the metropolitan microbiome, along with the potential for both honey bee and human pathogen surveillance^112^. A study discovered that the urban metagenome was a reservoir of novel microbial species, antimicrobial resistance markers, and CRIPSR arrays which could be a boon for further research applications^113^. This is a crucial reminder that ecological and biological surveys should also include metropolitan environments, with honey-based biosurveys containing the potential to monitor areas undergoing urbanisation or other critical environments where ecological transition may occur.

Although managed bee colonies may prove effective as agents for biomonitoring and biosurveys, we need to remember that bees are living creatures, which affords them the ethical considerations as any other domesticated animal^114^. In urban areas, where citizen scientists and hobbyist collaboratives may see an increase in beekeeping activities, we need to ascertain that those involved are aware of the safety risks from stinging, as well as have accountability for keeping and maintaining living creatures^115^. Furthermore, we need to ensure that managed bee populations do not negatively affect the ecological conditions of the areas they are introduced to, such as the introduction of disease to other insects or outcompeting endemic and native creatures for resources^115^. There has been evidence that the introduction of managed bee populations can negatively affect the abundance of wild bee and native pollinator species, meaning care needs to be taken when deploying managed bee colonies for biosurvey purposes^116,117^. This also indicates a need to look past domesticated honey bees and towards endemic or indigenous melliferous species when attempting to survey biodiversity within pristine or at-risk ecosystems.

Future biosurvey endeavours could feasibly extend to another melliferous flying (wasps, bumblebees and stingless bees)^118^ and non-flying insects (such as certain ant species)^119^, which could potentially expand the ecological niches that can be accessed, reducing the reliance on managed bees. Other flying-insect pollinators also appear to be at risk of decline^120^, and the DNA-based methods developed for the conservation effort for honey bees should also extend to other ecologically vital organisms. One of the key reasons identified for declines in alternative pollinators such as wild bees is habitat loss, as it leads to a shortage of floral resources^121,122^. This is of particular concern to bee species with narrow pollen diets^123^. The application of DNA analysis techniques with such pollinators would provide an instrument not only to monitor decreases in floral biodiversity, but to guide ecological efforts to restore habitats by identifying pollinators’ preferred floral species based on frequency of interaction.

## Conclusion

The worth of honey bees and their contribution across various industries and research efforts cannot be underestimated, with techniques intended for authentication and biosecurity containing the potential to elucidate diverse environments. The ability to detect a broad range of local biodiversity from a sample of honey may become a key tool in advancing geogenomics and conservation biogeography. The extensive foraging of honey bees and other melliferous insects serves to sample and condense genetic information from a wide ecological region. This approach may provide an avenue for improving spatiotemporal surveys of biodiversity, for which traditional methods have found difficulty in scaling. As cornerstone species in many environments, the collective genetic information provided by honey bees and their products provides a focal medium to understand ecological ties that drive vital ecosystem processes. This data can help build information on ecological networks and can be used to explain the evolution, distribution and symbiosis of the associated and integrated biodiversity of local ecosystems. Advances in next-generation sequencing technologies and in DNA-based analysis methods will drive metagenomics from a field that predominantly explores microorganisms to one that can also include non-microbial and multicellular eukaryotes, with honey providing the ideal substrate for plying this progress due to the expansive breadth of collective taxa contained within. The collective information in honey provides a trove of novel data for biotechnology and (micro)biology and can help drive conservation by monitoring global biodiversity via geogenomics and biogeography studies. This means that promoting future honey-based biosurveys will likely provide a sweet deal to industry, research, and conservation interests alike.
